# Investigation of the Relationship Between Serum Trace Elements and MSFC Levels in Multiple Sclerosis: A Cross-sectional Case–Control Study

**DOI:** 10.1007/s12011-025-04643-9

**Published:** 2025-05-05

**Authors:** Cihat Ozguncu, Serefnur Ozturk, Haluk Gumus, Gokhan Ozdemir, Bahadır Ozturk, Ilkay Guzel, Gozde Ongun

**Affiliations:** 1 Department of Neurology, Faculty of Medicine, Selcuk University, Akademi Mahallesi, Yeni İstanbul Caddesi No:313, 42130 Selcuklu, Konya, Turkey; 2 Department of Medical Biochemistry, Faculty of Medicine, Selcuk University, Konya, Turkey; 3 Department of Neurology, Medicalpark Hospital, Istanbul, Turkey

**Keywords:** Depression, Fatigue, Multiple sclerosis, Trace element, Chromium, Manganese

## Abstract

The effects of trace elements have been investigated in multiple sclerosis (MS) etiology for years. However, common results have yet to be obtained in previous studies. Therefore, as a cross-sectional case–control study, we aimed to examine the relationship between trace elements and MS in the Konya province, where air pollution is intense. Study participants with MS were investigated regarding the expanded disability status (EDSS) and multiple sclerosis functional composite (MSFC) scales. All study participants were also evaluated concerning the Beck Depression Inventory (BDI), Facit Fatigue Scale (FFS), Fatigue Severity Scale (FSS), and levels of blood chromium (Cr), manganese (Mn), copper (Cu), zinc (Zn), selenium (Se), cadmium (Cd), and lead (Pb). While there were 49 people in the MS group, 51 individuals constituted the controls. Mn, Zn, Cd, and Pb levels were significantly higher in controls. There was no significant difference between MS and control groups regarding depression and fatigue scores. MSFC parameters, depression, and fatigue scale scores were statistically higher with increasing EDSS in MS patients. A significant correlation was also observed between the scores of the timed 25-foot walk (T25FW) and dominant hand 9-hole peg test (9HPT) and Cr levels (*p* = 0.014, 0.004). A relationship was also observed between Mn levels and T25FW (*p* = 0.047). Trace element levels can be seen at different levels in MS patients. While a correlation was observed between some MSFC parameters and Cr and Mn levels, no relationship was observed between trace element levels, depression, fatigue, and EDSS.

## Introduction

Multiple sclerosis (MS) is a chronic disease that affects the central nervous system [1]. It causes inflammation, demyelination, and axon loss. Nearly 3 million people worldwide are living with MS [2]. Depending on the location of the inflammation, different symptoms may accompany it. Patients may experience symptoms such as vision loss, weakness, fatigue, bladder and bowel control problems, pain, imbalance, cognitive dysfunction, muscle strength loss, and walking difficulty/loss [3]. The incidence is concentrated between the ages of 20 and 40. MS occurs more frequently in women. It deserves special attention, mainly because it is an important cause of morbidity at a young age [4].

After the identification of MS, various studies have commenced investigating the etiology of MS rapidly. Although environmental, genetic, and autoimmune factors are emphasized to be effective in its etiology, how MS starts and what causes the progression has yet to be clearly understood [5]. Therefore, a definitive treatment for the disease has not yet been found. Regarding environmental factors, mining areas, active volcano regions, and industrial zones with intense air pollution are rich in heavy metals and trace elements [6]. The accumulation of heavy metals and trace elements is known to be directly related to various disease groups. Different heavy metals and trace elements can lead to diseases by acting through different pathways or play an important role in cellular functions by participating in the structure of enzymes. In case of heavy metal imbalance in the cerebrospinal fluid (CSF), the condition can lead to pathological lesions involving the blood–brain barrier, accumulation of heavy metals, and neurodegeneration [7].

Zinc (Zn) is vital in regulating the immune system, showing its effect through the tumor necrosis factor (TNF)-alpha inhibitors. The deficiency of Zn can lead to the weakness of the immune system and contributes to the pathogenesis of MS by causing an imbalance between the functions of T helper-1 (Th1) and T helper-2 (Th2) cells and the failure of Th17 downregulation [8]. Zn is also important in neurogenesis, neurotransmitter activity, and the basic protein of myelin. For all these reasons, it can be said that zinc has an important place in the pathogenesis of MS [9, 10]. Copper (Cu) plays a key role in the synthesis of myelin. The deficiency of Cu can potentially lead to myelinopathy, and Cu also shows its effectiveness by binding to vital enzymes such as cytochrome oxidase and superoxide dismutase [11]. In addition, when the CSF of MS patients was examined, the levels of Cu were observed to be significantly increased compared to the normal population [12]. After exposure to lead (Pb), the production of autoantibodies was observed against the proteins of the central nervous system (CNS), such as myelin basic protein (MBP) and glial fibrillary acidic protein (GFAB). It is estimated that these antibodies play a role in demyelination and, therefore, in the pathogenesis of MS. [13]. Given that cadmium (Cd) is known as a neurotoxic metal upon exposure to high levels, polyneuropathy is observed, and Cd is also considered to have effects on the blood–brain barrier [14, 15]. It is stated that cadmium may play a role in the pathogenesis of MS through oxidative stress or increased lipid peroxidation [16]. Another element, selenium (Se), is a trace element found in a narrow range in the body. In studies, Se has been suggested to be associated with the neurodegenerative diseases of CNS [17]. Additionally, Se can also bring on mitochondrial damage by leading to the formation of reactive oxygen radicals and inhibiting antioxidant reactions. It is anticipated that the effects of Se may lead to neuro-toxication [18, 19]. It is suggested that selenium has important functions in neuronal signaling pathways and affects motor and cognitive functions [20, 21]. However, the possible role of selenium in MS pathogenesis is controversial in the literature. While some studies indicate that it has a place in pathology, there are also studies suggesting that it has no place in MS pathology [21–23]. Acting as a cofactor in many enzymes, manganese (Mn) is another trace element known to have important functions in the proper functioning of the immune system [21, 24]. Although the role of manganese in MS pathophysiology is unknown, it is stated that neuronal physiology and cognition are affected by the dysregulation of Mn levels [25]. Finally, chromium (Cr) is a rare transition metal, and it has been reported that Cr can be stored in the body and may cause neurotoxicity [26].

Such features as its geographical structure spreading on a broad plateau in Central Anatolia, climate, and proximity to the industrial zones are among the factors causing the province of Konya to rank first in Turkey regarding air pollution [27]. Our study aimed to compare the levels of some trace elements (Cr, Mn, Cu, Zn, Pb, Se, Cd) in the serum of MS patients with the control group and to examine the relationship between these trace elements and disease severity (EDSS), functionality and cognitive skills (MSFC), and depression and fatigue in individuals with MS.

## Methods

### Participants

Our study was designed as a single-center cross-sectional case–control study. Our study was performed between August and November 2021 with approval from the Local Ethics Committee of the Faculty of Medicine at Selcuk University (Reg. number: 2019/81). To test a two-sided hypothesis regarding whether there is a significant difference between the trace element levels of MS patients and control group individuals with a 5% significance level (type-1 error), a large effect size (Cohen’s *d* = 0.8), and 80% statistical power using an independent samples *t*-test, it was calculated with a priori power analysis that at least 52 individuals in total, at least 26 in each arm, should be included in the study. Patients over 18 years of age, diagnosed with MS under the 2017 McDonald criteria [28], and those with no rheumatological disease, pregnancy, and not being in an attack period were included in the study after obtaining informed written consent. Participants included in the study were expected to speak Turkish fluently. The study did not include individuals with psychiatric disorders or cognitive dysfunction that could affect cooperation. Patients with a disease other than multiple sclerosis that affected hand function (such as peripheral nerve lesions, previous trauma/surgery, or cerebrovascular disease) were not included in the study. Patients with heart failure and/or lung pathology that could affect performance in daily activities were omitted. For all patients, such features as age, gender, comorbid diseases, duration and subtype of MS, medications administered to treat MS, living in proximal homes to the city center, frequency of fish consumption, smoking status, and educational status were recorded.

To avoid possible errors and confusion, a single neurologist who received training and certification from Neurostatus, an independent platform that aims to standardize the expanded disability status scale (EDSS) [29] measurement, performed EDSS and multiple sclerosis functional composite (MSFC) tests on all patients. All tests and examinations were performed in a session lasting approximately 1–1.5 h. The timed 25-foot walk test (T25 FW), 9-hole peg test (9HPT), and paced auditory serial addition test (2-s and 3-s versions) (PASAT2 and PASAT3) tests included in the MSFC were administered to MS patients [30].

MS-specific scales such as FSIQ-RMS were developed by Ruotolo and colleagues for fatigue [31], which is one of the most common complaints in MS patients from the early stages [32]. However, we decided to use more general scales since we had to apply the same scale to the control and patient groups. The Fatigue Severity Scale (FSS), developed by Krupp et al. and whose Turkish validity and reliability study was conducted by Armutlu et al., was applied to the entire study population. A score of 36 > on FSS was evaluated as fatigue [33, 34]. Similarly, the FACIT-Fatigue Scales (FFS), developed by Webster et al. and adapted to MS by Delgado-Alverez et al., was applied. [35, 36]. In addition, the Beck Depression Inventory (BDI), which is widely used to assess depression worldwide and developed by Beck et al. and whose Turkish validity and reliability were tested by Kapçi et al., was recorded [37, 38]. A score of 17 or higher acquired in BDI was considered depression. The patients completed the BDI, FSS, and FFS scales themselves.

**Fig. 1:**
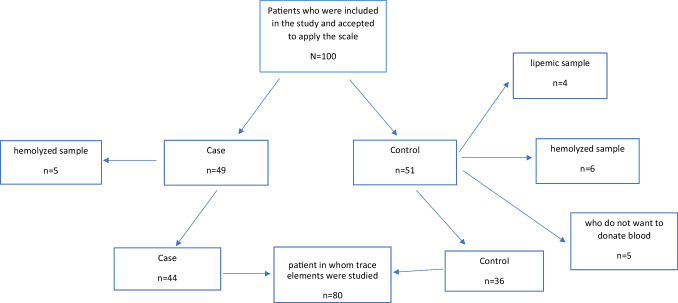
Participant flow diagram

Patients over 18 who applied to the neurology clinic with simple neurological complaints (such as headache and dizziness) were included in the study as a control group. When selecting the control group, individuals with known chronic diseases, continuous medication use, psychiatric diseases, rheumatological diseases, and pregnancies were excluded from the study. Informed written consent was obtained from all participants. When selecting the control group, care was taken to ensure that the gender ratio and age were similar to the MS patient population. In the control group, the features such as age, gender, distance from the homes to the city center, smoking status, and fish consumption were recorded, and the scales of BDI, FSS, and FFS were also carried out in the control group. A total of 100 participants agreed to take part in the study and completed the scales. However, blood samples from five individuals in the MS group were hemolyzed, preventing the analysis of their trace element levels. Trace element levels were successfully measured and recorded for 44 patients in this group. In the control group, six blood samples were hemolyzed, and four samples were lipemic. Additionally, five participants chose not to donate blood later but remained in the study. Consequently, trace element levels were measured for 36 individuals from the control group. Participants who did not provide a blood sample were not excluded from the study, and their completed scales were included in the analysis. Since our priori power analysis indicated that 26 patients in each group would be sufficient, no new participants were recruited for the study. A participant flow diagram of the study was created (Fig. 1).

### Analysis of Trace Elements

The blood sample taken from the study population was transferred to BD vacutainer K2EDTA (10.8 mg) trace element tubes by venous route. It was left to clot for 15–30 min at room temperature. The serum obtained by centrifuging at 5000 rpm for 5 min was transferred to an Eppendorf tube and stored at − 80 °C until examination. After the samples were collected, they were sent to the advanced technology laboratory affiliated with our university. One-milliliter samples were taken from the tubes. First, organic matter was separated with 2 ml of hydrogen peroxide. It was diluted tenfold with 5 ml of nitric acid solution. The samples were then taken into the microwave and burned at 160 °C for 25 min after a 15-min ramp period (100% power 1600 W). The samples remained in the microwave for 40 min (CEM MARS5, CEM Corporation, Germany). Zn, Mn, Cu, Pb, Se, Cr, and Cd levels were measured by inductively coupled plasma mass spectrometry (ICP-MS) with 9-point calibration (*R* > 0.999) (1–10–25–50–100–250–500–1000-2000) covering the 1–2000 ppm level (Perkin Elmer/ICP MS ELAN DRC-E, PerkinElmer Inc, Wellesley, USA). Perkin Elmer N930033 calstt3 was used as the standard material for calibration.

### Statistical Analysis

All statistical analysis was performed using the R version 3.6.0 (The R Foundation for Statistical Computing, Vienna, Austria; https://www.r-project.org). Shapiro–Wilk’s normality test and Q-Q plots were used to assess the normality of the data. Also, the Levene test was performed to check the homogeneity of the variances. The numerical variables were presented as mean ± standard deviation (SD) (range: minimum–maximum) or median (interquartile range [IQR]: 25 th percentile–75 th percentile) as appropriate. Even so, the categorical variables were described as number (*n*) and percentage (%). T25 FW was converted to *Z* score as Z-LEG to calculate multiple sclerosis functional composite. Similarly, 9HPT was converted to Z-ARM, and PASAT-3 was converted to Z-COG. MSFC score was calculated with Z-ARM, Z-LEG, and Z-COG [39]. Reference values from the literature were used during the calculation [30]. An independent samples *t*-test, Welch’s *t*-test, the Mann–Whitney *U* test, the Yates continuity correction chi-square test, and the Fisher-Freeman-Halton test were conducted to determine whether there was a statistically significant difference or association between the study groups regarding the demographical and clinical characteristics and laboratory findings. The Spearman-rho ranked-order correlation coefficients were used to examine the relationship between numerical variables. Multivariate logistic regression analysis was performed for significant parameters. A two-tailed *p*-value of < 5% was considered statistically significant.

## Results

While there were 49 patients with a mean age of 35.08 ± 11.36 years in the MS group, the control group was composed of 51 individuals with a mean age of 31.41 ± 10.71 years. The male-to-female (M/F) ratios were 32/17 and 31/20 in the MS and control groups, respectively. The mean duration of MS was detected as 5 years (1–27), and the mean EDSS score was calculated as 2.81 (0.5–5.5). The distributions of the MS subtypes among the patients were as follows: 41 patients with relapsing–remitting multiple sclerosis (RRMS), four with primary progressive multiple sclerosis (PPMS), and four with secondary progressive multiple sclerosis (SPMS). The findings are presented in Table 1.

**Table 1:** Demographical and clinical characteristics of multiple sclerosis patients and controls

	**MS, *n* (%)**	**Controls, *n* (%)**	***p*-value**
**Demographical characteristics**			
Age (years)	35.08 ± 11.36	31.41 ± 10.71	.100^1^
Gender (F/M)	32 (65.3)/17 (34.7)	31 (60.8)/20 (39.2)	.794^2^
**Duration of MS**	5 (1–27)		
**MS scores**			
EDSS	2.81 ± 1.58 (0.5–5.5)		
T25 FW	7.17 ± 2.70 (4.4–16.1)		
9HPT dominant	23.39 ± 8.70 (13.9–52.8)		
9HPT non-dominant	27.19 ± 11.29 (16–58.3)		
PASAT 3-Seconds Version	27.67 ± 5.57 (9–48)		
PASAT 2-Seconds Version	25.54 ± 6.71 (2–43)		
Z-ARM	0.02 ± 1.11 (− 2.53 to 2.31)		
Z-LEG	0.21 ± 0.24 (− 0.58 to 0.45)		
Z-COGNITIVE	− 1.48 ± 0.56 (− 3.73 to 0.25)		
MSFC	− 0.42 ± 0.53 (− 1.77 to 0.46)		
**Type of MS**			
RRMS	41 (83.7)		
SPMS	4 (8.2)		
PPMS	4 (8.2)		
**Drugs administered**			
Glatiramer acetate	1 (2)		
Interferon beta-1a	5 (10.2)		
Teriflunomide	2 (4.1)		
Dimethyl fumarate	8 (16.3)		
Fingolimod	13 (26.5)		
Ocrelizumab	10 (20.4)		
Rituximab	2 (4.1)		
Cladribine	2 (4.1)		
No drugs	6 (12.2)		

*EDSS* expanded disability status scale, *MS* multiple sclerosis, *9HPT* dominant hand 9-hole peg test, *PASAT* paced auditory serial addition test, *PPMS* primary progressive multiple sclerosis, *RRMS* relapsing–remitting multiple sclerosis, *SPMS* secondary progressive multiple sclerosis, *T25 FW* timed 25-foot walk test, *MSFC* Multiple Sclerosis Functional Composite

The levels of Mn, Zn, Cd, and Pb were significantly higher in the control group (*p* < 0.001, 0.002, < 0.001, < 0.001, respectively). There was no significant difference between the MS and control groups in terms of depression and fatigue scores, and the comparisons are given in Table 2.

**Table 2:** Comparisons between multiple sclerosis patients and controls in the study

	**MS, *n* (%)**	**Controls, *n* (%)**	***p*-value**
**Laboratory findings**			
Cr	733.66 ± 157.24	725.10 ± 217.60	.839^1^
Mn	26.59 (22.84–42.13)	52.91 (35.80–74.96)	<.001^2^
Cu	1708 ± 388.58	1699.39 ± 447.40	.927^1^
Zn	868.96 ± 190.56	1067.38 ± 322.79	.002^3^
Se	159.71 ± 74.85	155.79 ± 26.78	.518^1^
Cd	0 (0–0.03)	0.17 (0–0.39)	<.001^2^
Pb	7.5 (0–25.55)	28 (17.08–65.5)	<.001^2^
**Scales**			
BDI	17.39 ± 12.88	13.55 ± 9.59	.098^3^
Beck (≥ 17)	22 (44.9)	16 (32.7)	.300^4^
FFS	23.47 ± 13.83	21.29 ± 10.06	.374^3^
FSS	41.70 ± 18.30	36.18 ± 14.82	.109^1^
FSS (≥ 36)	30 (65.2)	26 (53.1)	.320^4^
**Smoking status**	13 (26.5)	22 (43.1)	.126^4^
**Fish consumption**			.893^5^
About 1 per week	5 (10.2)	7 (13.7)	
About 1 per month	29 (59.2)	30 (58.8)	
About 1 per year	13 (26.5)	11 (21.6)	
None	2 (4.1)	3 (5.9)	
**Residence area**			.036^5^
Province	38 (77.6)^a^	48 (94.1)^b^	
County	9 (18.4)	3 (5.9)	
Others	2 (4.1)	0 (0)	
**Employment status**			.005^4^
Employed	17 (34.7)	33 (64.7)	
Unemployed	32 (65.3)	18 (35.3)	

Data were expressed as mean ± standard deviation (SD), median (interquartile range: 25 th percentile–75 th percentile), or number (*n*) and percentage (%). ^1^Independent samples *t-*test, ^2^Mann-Whitney *U* test, ^3^Welch’s *t*-test, ^4^Yates continuity correction chi-square test, ^5^Fisher-Freeman-Halton test. *BDI* Beck Depression Inventory, *FFS* Facit Fatigue Scale, *FSS* Fatigue Severity Scale, *Cd* cadmium, *Cr* chromium, *Cu* copper, *Mn* manganese, *MS* multiple sclerosis, *Pb* lead, *Se* selenium, *Zn* zinc

Given the evaluation of MSFC of MS patients, the parameters of MSFC and depression and fatigue scales were observed to be statistically higher, as was the increase in EDSS. A significant correlation was also observed between T25 FW and 9HPT scores and Cr levels (*p* = 0.014, 0.004, respectively). Similarly, a significant correlation was observed between manganese level and T25 FW and *Z* score (*p* = 0.047) (Table 3).

**Table 3:** Evaluation of the relationship between the scales in patients with multiple sclerosis

	**EDSS**	**T25 FW**	**9HPT dominant**	**9HPT non-dominant**	**PASAT 3**	**Z-ARM**	**Z-LEG**	**Z-COG**	**MSFC**
T25 FW	**0.866** ** < 0.001**	–	–	–	–				
9HPT dominant	0.763* ** < 0.001**	0.782* ** < 0.001**	–	–	–				
9HPT non-dominant	0.738* ** < 0.001**	0.678* ** < 0.001**	0.690* ** < 0.001**	–	–				
PASAT 3-Seconds Version	0.446* ** < 0.001**	0.526* ** < 0.001**	0.516* ** < 0.001**	0.360* **0.012**	–				
PASAT 2-Seconds Version	0.364* **0.011**	0.454* ** < 0.001**	0.486* ** < 0.001**	0.363* **0.011**	0.857* ** < 0.001**	0.857* ** < 0.001**	0.454* **0.001**	0.857* ** < 0.001**	0.630* ** < 0.001**
BDI	0.551* ** < 0.001**	0.491* ** < 0.001**	0.491* ** < 0.001**	0.512* ** < 0.001**	0.295* **0.042**	− 0.585* ** < 0.001**	− 0.514* ** < 0.001**	− 0.283* **0.049**	− 0.582* ** < 0.001**
FFS	0.697* ** < 0.001**	0.609* ** < 0.001**	0.509* ** < 0.001**	0.560* ** < 0.001**	0.287* **0.048**	− 0.410* **0.005**	− 0.519* ** < 0.001**	− 0.104 0.479	− 0.496* ** < 0.001**
FSS	0.643* ** < 0.001**	0.493* ** < 0.001**	0.399* **0.006**	0.447* **0.002**	− 0.161 0.289	** − 0.410** **0.005**	− 0.408* **0.005**	− 0.15 0.923	− 0.352* **0.016**
Level of Cr	0.187 0.199	0.368* **0.014**	0.426* **0.004**	0.154 0.291	0.024 0.869	− 0.141 0.335	− 0.006 0.965	0.220 0.129	− 0.020 0.893
Level of Mn	0.176 0.253	0.301* **0.047**	0.057 0.711	0.010 0.951	− 0.188 0.222	− 0.08 0.606	− 0.301* **0.047**	− 0.273 0.073	− 0.192 0.211
Z-ARM	− 0.801* ** < 0.001**	− 0.685* ** < 0.001**	− 0.890* ** < 0.001**	− 0.886* ** < 0.001**	0.471* **0.001**	–	–	–	–
Z-LEG	0.791* ** < 0.001**	− 1* ** < 0.001**	− 0.607* ** < 0.001**	− 0.525* ** < 0.001**	0.526* ** < 0.001**	0.685* ** < 0.001**	–	–	–
Z-COG	− 0.400* **0.004**	− 0.476* **0.001**	− 0.313* **0.028**	− 0.221 0.127	1	0.368* **0.009**	0.476* **0.001**	–	–
MSFC	− 0.817* ** < 0.001**	− 0.791* ** < 0.001**	− 0.818* ** < 0.001**	− 0.771* ** < 0.001**	0.684* ** < 0.001**	0.924* ** < 0.001**	0.791* ** < 0.001**	0.681* ** < 0.001**	–

*BDI* Beck Depression Inventory, *Cr* chromium, *EDSS* expanded disability status scale, *FFS* Facit Fatigue Scale, *FSS* Fatigue Severity Scale, *9HPT* dominant hand 9-hole peg test, *PASAT* paced auditory serial addition test, *T25 FW* timed 25-foot walk test, *Z-COG* cognitive *Z* score, *MSFC* Multiple Sclerosis Functional Composite, *Cr* cromium, *Mn* manganese

In addition, the values of the elements, + Mn (26.59 [IQR, 22.84–42.13] vs. 52.91 [IQR, 35.80–74.96], *p* < 0.001), Zn (868.96 ± 190.56 vs. 1067.38 ± 322.79, *p* = 0.002), Cd (0 [IQR, 0–0.03] vs. 0.17 [IQR, 0–0.39], *p* < 0.001), and Pb (7.5 [IQR, 0–25.55] vs. 28 [IQR, 17.08–65.5], *p* < 0.001) were determined to be lower in the MS patient group, compared to the healthy controls.

When the correlations among trace elements were evaluated, it was observed that there was a significant correlation between Mn level and Cr, Cd, and Pb (*p* = 0.021, < 0.001, < 0.001, respectively), a significant correlation between Se levels and Cr, Zn, and Cd (*p* = 0.002, 0.014, 0.048, respectively), a correlation between Cr and Cu, and a correlation between Cd and Pb (*p* = 0.012, < 0.001, respectively) (Table 4). The logistic regression analysis observed that the significance between trace elements and MSFC parameters continued.

**Table 4:** Evaluation of the relationship between the trace elements

	Cr	Mn	Cu	Zn	Se	Cd
Mn	0.258* **0.021**	–				
Cu	0.280* **0.012**	0.034 0.768	–			
Zn	− 0.101 0.372	0.194 0.085	0.168 0.137	–		
Se	0.342* **0.002**	− 0.102 0.366	0.213 0.058	0.275* **0.014**	–	
Cd	− 0.051 0.650	0.414* ** < 0.001**	− 0.123 0.275	0.146 0.197	− 0.222* **0.048**	-
Pb	− 0.130 0.252	0.503* ** < 0.001**	− 0.210 0.061	0.117 0.301	− 0.194 0.085	0.580* ** < 0.001**

*Mn* manganese, *Cu* copper, *Zn* zinc, *Se* selenium, *Cd* cadmium, *Pb* lead, *Cr* cromium

## Discussion

Although many studies are investigating the association between MS and heavy metals based on the literature, various points remain unexplained, and studies report conflicting findings regarding the relationship between MS and heavy metals. The relationship between the severity of MS and exposure to higher levels of heavy metals is one of the challenges to be clarified.

In light of the literature, in a systematic review evaluating the effects of heavy metals on MS, it was reported that no difference was observed between the MS and control groups regarding Se and Cu levels, and Zn was found lower, as consistent with our study findings. On the other hand, the Mn levels were higher in MS patients [21]. In a similar meta-analysis, the levels of Cd were stated to be higher in MS patients. Although the levels of Pb were higher in MS patients, the difference was considered statistically insignificant [40]. Another meta-analysis evaluating only the Zn levels showed that the Zn levels were significantly lower in the MS population than in the controls, which aligns with our study results [41].

In two different studies conducted in Iran, where air pollution is intense, as in the province of Konya, the levels of Pb were found to be similar between the MS patient and control groups. Another study also evaluated the levels of Cu and Se within patients with MS. It detected that the levels of both elements were at a similar rate between the MS and control groups, consistent with our study findings [10]. In the study carried out by Aliomrani et al., the level of Cd was determined to be higher in the MS group [42]. In a study published in 2017 that included findings similar to our study, the levels of Cd, Zn, and Mn were observed to be decreased in MS patients compared to those in the control group [43]. A similar study recently showed that MS patients had lower Zn and Mn levels than controls, as in our study [44]. However, a meta-analysis by the same authors concluded that Mn levels were not significantly lower. Zn levels were reported to be lower in the MS group, as in our study [1]. We believe that Mn levels in MS patients deserve re-evaluation with more patients. In the literature, there are also results complying with our study findings, revealing that the levels of Pb and Zn are lower in MS patients [45]. In a study carried out in Brazil and including completely different findings from our study, it was stated that the levels of Cu and Cr were lower, the level of Pb was higher, and there was no difference concerning Zn levels in MS patients [46]. Such differences suggested that the results in different studies could vary considerably according to regions and geographical features where the studies were conducted.

Although the results in the literature and our study are generally compatible, some studies report conflicting results regarding the levels of Pb and Cd in MS patients. It is unlikely that any comments will be made about whether such a situation plays a role as a by-product in the pathophysiology of MS but has no direct effect on the issue or whether MS has an unknown mechanism yet. Although not statistically significant, it was revealed that the rate of smoking in the control group was relatively high and that they lived more in the city center. Such factors may have caused abnormalities in heavy metal accumulation. It is stated that Cd and Pb levels are higher in people with smoking habits compared to controls [47].

Our study observed no significant relationship between fish consumption, smoking habits, distance to the city center, where air pollution is high, and disability. However, it is stated in the literature that the progression of the disease can be affected by changing environmental and nutritional factors [48]. Our study observed that, along with the increase in EDSS, the parameters of MSFC, depression, and fatigue scales were also statistically higher. Those findings are supported by more than one study in the literature [49, 50]. Appropriate rehabilitation practices can also somewhat reverse MSFC parameters [51]. In many studies, it is stated that there is an increase in depression and fatigue scores in MS patients compared to the controls [52, 53]. Even so, an increase was observed in the depression and fatigue scores in our MS patients; the difference displayed no statistical significance. Therefore, it is likely to make different inferences, and we consider that the entity deserves further evaluation. Although our study revealed a statistically significant relationship between the scores of T25 FW and 9HPT dominant and the levels of Cr, no publications supporting or contradicting such a finding were encountered in the literature. The levels of Cr were within normal limits in our samples, and there was no finding suggestive of toxicity. A placebo-controlled double-anonymized study investigating the effects of Cr supplementation on neuronal functions reported that it could increase cognitive inhibitory control and cerebral function in older adults at risk for neurodegeneration [54]. Similarly, a meta-analysis reported lower chromium levels in children with cognitive deficits [55]. Of course, although these studies did not evaluate the effects of chromium in MS patients, they provide an idea about the effect of chromium on cognitive function. Further studies should explain the necessity of Cr supplementation in MS patients and its importance on cognitive and motor functions.

In our study, a correlation is observed between T25 FW and Mn levels. No publication in the literature clearly explains the effect of Mn levels on walking function. However, publications indicate that cows whose diets contain trace elements (zinc, manganese, copper, and cobalt) positively increase their kinematic walking parameters. In this publication, it was reported that no difference was observed between the zinc and copper levels in the blood of the control group and the experimental group. However, there was a significant increase in manganese levels in those receiving supplementary food [56]. More research needs to be done on this issue.

The present study also has several limitations. First, no national and geographical characteristics could be evaluated since the data obtained from a single center were utilized. This situation limits the generalization of the findings. Secondly, the levels of heavy metals and trace elements evaluated in the study may be affected by many clinical and structural conditions; in other words, nutritional supplements and lifestyle habits can alter the levels of serum elements. Additionally, no occupational details were evaluated in the population studied; some occupations are known to be risky due to exposure to heavy metals. One of the most important limitations of the study is that it included a small number of cases and controls. Finally, although depression and fatigue are known to be affected by many conditions, those conditions have been ignored. It is recommended that those conditions be considered and investigated in studies that include more significant populations.

As a result of the findings in our study and previous studies, it still seems unlikely that a clear relationship between MS and the levels of heavy metals and trace elements can be established. However, the lower levels of certain elements, especially in MS patients, support the hypothesis that those elements and their levels may be a distinguishing factor in MS. In particular, the relationship between Cr and Mn levels and MSFC parameters needs to be re-evaluated and confirmed by further studies. With novel tests and methods, the etiology and pathophysiology of MS will be enlightened more clearly, and the question marks will be eliminated as a result of such studies.

## Conclusion

The Mn, Zn, Cd, and Pb levels were lower in the MS group compared to the control, while the Se, Cr, and Cu levels were similar. No correlation was observed between trace element levels and depression, fatigue, and EDSS scores. A significant relationship was observed between EDSS and MSFC parameters and depression and fatigue scores. A significant correlation was observed between Cr and Mn levels and some MSFC parameters.

## Data Availability

The raw data supporting the outcomes of this article will be made available by the authors without undue reservation.

## References

[1] Stojsavljević A., et al., Changes of target essential trace elements in multiple sclerosis: a systematic review and meta-analysis. Biomedicines, 2024. **12**(7). 10.3390/biomedicines12071589 10.3390/biomedicines12071589 PMC11274787 39062163

[2] Filippi M. and M.A. Rocca, Multiple sclerosis, in white matter diseases : an update for neurologists. 2020, Springer International Publishing: Cham. p. 1–35. 10.1007/978-3-030-38621-4_1

[3] Ghasemi N, Razavi S, Nikzad E (2017) Multiple sclerosis: pathogenesis, symptoms, diagnoses and cell-based therapy. Cell J 19(1):1–10. 10.22074/cellj.2016.4867 28367411 10.22074/cellj.2016.4867 PMC5241505

[4] Harbo HF, Gold R, Tintoré M (2013) Sex and gender issues in multiple sclerosis. Ther Adv Neurol Disord 6(4):237–248. 10.1177/1756285613488434 23858327 10.1177/1756285613488434 PMC3707353

[5] Kamińska J et al (2017) Multiple sclerosis - etiology and diagnostic potential. Postepy Hig Med Dosw (Online) 71:551–563. 10.5604/01.3001.0010.3836 28665284 10.5604/01.3001.0010.3836

[6] Hansell, A.L., C.J. Horwell, and C. Oppenheimer, The health hazards of volcanoes and geothermal areas. Occup Environ Med, 2006. **63**(2): p. 149–56, 125. 10.1136/oem.2005.022459 10.1136/oem.2005.022459 PMC2078062 16421396

[7] Madeddu R et al (2011) Heavy metals and multiple sclerosis in sardinian population (Italy). Anal Lett 44(9):1699–1712. 10.1080/00032719.2010.520396

[8] Mezzaroba L et al (2019) The role of zinc, copper, manganese and iron in neurodegenerative diseases. Neurotoxicology 74:230–241. 10.1016/j.neuro.2019.07.007 31377220 10.1016/j.neuro.2019.07.007

[9] Li, Z., et al., The ımportant role of zinc in neurological diseases. Biomolecules, 2022. **13**(1). 10.3390/biom13010028 10.3390/biom13010028 PMC9855948 36671413

[10] Alizadeh, A., et al., Comparison of serum concentration of Se, Pb, Mg, Cu, Zn, between MS patients and healthy controls. Electron Physician, 2016. **8**(8): p. 2759–2764. 10.19082/2759 10.19082/2759 PMC5053457 27757186

[11] De Riccardis, L., et al., Copper and ceruloplasmin dyshomeostasis in serum and cerebrospinal fluid of multiple sclerosis subjects. Biochimica et Biophysica Acta (BBA) - Molecular Basis of Disease, 2018. 1864(5, Part A): p. 1828–1838. 10.1016/j.bbadis.2018.03.007 10.1016/j.bbadis.2018.03.007 29524632

[12] Melø TM et al (2003) Manganese, copper, and zinc in cerebrospinal fluid from patients with multiple sclerosis. Biol Trace Elem Res 93(1):1–8. 10.1385/BTER:93:1-3:1 12835484 10.1385/BTER:93:1-3:1

[13] El-Fawal, H.A., et al., Neuroimmunotoxicology: humoral assessment of neurotoxicity and autoimmune mechanisms. Environ Health Perspect, 1999. **107 Suppl 5**(Suppl 5): p. 767–75. 10.1289/ehp.99107s5767 10.1289/ehp.99107s5767 PMC1566239 10502543

[14] Viaene MK et al (1999) Cadmium: a possible etiological factor in peripheral polyneuropathy. Neurotoxicology 20(1):7–16 10091854

[15] Méndez-Armenta M et al (2003) Brain regional lipid peroxidation and metallothionein levels of developing rats exposed to cadmium and dexamethasone. Toxicol Lett 144(2):151–157. 10.1016/S0378-4274(03)00199-1 12927359 10.1016/s0378-4274(03)00199-1

[16] Kooshki A et al (2024) Essential and toxic metal concentrations in biological samples of multiple sclerosis patients: a systematic review and meta-analysis. PLoS ONE 19(12):e0313851. 10.1371/journal.pone.0313851 39642137 10.1371/journal.pone.0313851 PMC11623488

[17] Filippini T, et al., Selenium neurotoxicity and amyotrophic lateral sclerosis: an epidemiologic perspective, in Selenium, B. Michalke, Editor. 2018, Springer International Publishing: Cham. p. 231–248. 10.1007/978-3-319-95390-8_12

[18] Oliveira CS, et al., Chemical speciation of selenium and mercury as determinant of their neurotoxicity, in neurotoxicity of metals, M. Aschner and L.G. Costa, Editors. 2017, Springer International Publishing: Cham. p. 53–83. 10.1007/978-3-319-60189-2_4 10.1007/978-3-319-60189-2_4 28889263

[19] Mandrioli J et al (2017) Elevated levels of selenium species in cerebrospinal fluid of amyotrophic lateral sclerosis patients with disease-associated gene mutations. Neurodegener Dis 17(4–5):171–180. 10.1159/000460253 28478440 10.1159/000460253

[20] Ding W et al (2023) Selenium and human nervous system. Chin Chem Lett 34(7):108043. 10.1016/j.cclet.2022.108043

[21] Nirooei E et al (2022) Blood trace element status in multiple sclerosis: a systematic review and meta-analysis. Biol Trace Elem Res 200(1):13–26. 10.1007/s12011-021-02621-5 33611740 10.1007/s12011-021-02621-5

[22] Socha K et al (2014) Dietary habits and selenium, glutathione peroxidase and total antioxidant status in the serum of patients with relapsing-remitting multiple sclerosis. Nutr J 13:62. 10.1186/1475-2891-13-62 24943732 10.1186/1475-2891-13-62 PMC4080729

[23] Rahmani M et al (2024) Shining a light on selenium: a meta-analysis of supplementation in multiple sclerosis. Biol Trace Elem Res 202(10):4375–4386. 10.1007/s12011-023-04026-y 38155333 10.1007/s12011-023-04026-y

[24] Erikson KM, Aschner M (2003) Manganese neurotoxicity and glutamate-GABA interaction. Neurochem Int 43(4):475–480. 10.1016/S0197-0186(03)00037-8 12742094 10.1016/s0197-0186(03)00037-8

[25] Balachandran RC et al (2020) Brain manganese and the balance between essential roles and neurotoxicity. J Biol Chem 295(19):6312–6329. 10.1074/jbc.REV119.009453 32188696 10.1074/jbc.REV119.009453 PMC7212623

[26] Wise JP et al (2022) Current understanding of hexavalent chromium [Cr(VI)] neurotoxicity and new perspectives. Environ Int 158:106877. 10.1016/j.envint.2021.106877 34547640 10.1016/j.envint.2021.106877 PMC8694118

[27] Yahaya NZ et al (2022) Air quality status in Konya City Centre, Konya, Turkey during pandemic Covid-19. IOP Conf Ser Earth Environ Sci 1013(1):012006. 10.1088/1755-1315/1013/1/012006

[28] Thompson AJ et al (2018) Diagnosis of multiple sclerosis: 2017 revisions of the McDonald criteria. The Lancet Neurology 17(2):162–173. 10.1016/S1474-4422(17)30470-2 29275977 10.1016/S1474-4422(17)30470-2

[29] Kurtzke JF (1983) Rating neurologic impairment in multiple sclerosis. Neurology 33(11):1444–1444. 10.1212/WNL.33.11.1444 6685237 10.1212/wnl.33.11.1444

[30] Cutter GR et al (1999) Development of a multiple sclerosis functional composite as a clinical trial outcome measure. Brain 122(5):871–882. 10.1093/brain/122.5.871 10355672 10.1093/brain/122.5.871

[31] Ruotolo I et al (2022) Italian translation and validation of fatigue symptoms and impacts questionnaire in relapsing multiple sclerosis (FSIQ-RMS). Neurol Sci 43(8):4925–4932. 10.1007/s10072-022-06080-1 35451663 10.1007/s10072-022-06080-1

[32] Sellitto G et al (2021) Outcome measures for physical fatigue in individuals with multiple sclerosis: a systematic review. Expert Rev Pharmacoecon Outcomes Res 21(4):625–646. 10.1080/14737167.2021.1883430 33504225 10.1080/14737167.2021.1883430

[33] Krupp LB., et al., The fatigue severity scale. Application to patients with multiple sclerosis and systemic lupus erythematosus. Arch Neurol, 1989. **46**(10): p. 1121–3. 10.1001/archneur.1989.00520460115022 10.1001/archneur.1989.00520460115022 2803071

[34] Armutlu K et al (2007) The validity and reliability of the Fatigue Severity Scale in Turkish multiple sclerosis patients. Int J Rehabil Res 30(1):81–85. 10.1097/MRR.0b013e3280146ec4 17293726 10.1097/MRR.0b013e3280146ec4

[35] Delgado-Álvarez A et al (2022) Validation of two new scales for the assessment of fatigue in multiple sclerosis: F-2-MS and FACIT-F. Multiple Sclerosis Related Disorders 63:103826. 10.1016/j.msard.2022.103826 35487033 10.1016/j.msard.2022.103826

[36] Webster K, Cella D, Yost K (2003) The Functional Assessment of Chronic Illness Therapy (FACIT) Measurement System: properties, applications, and interpretation. Health Qual Life Outcomes 1:79. 10.1037/t77273-000 14678568 10.1186/1477-7525-1-79 PMC317391

[37] Beck AT et al (1996) Comparison of Beck Depression Inventories -IA and -II in psychiatric outpatients. J Pers Assess 67(3):588–597. 10.1207/s15327752jpa6703_13 8991972 10.1207/s15327752jpa6703_13

[38] Kapci EG et al (2008) Beck Depression Inventory II: evaluation of the psychometric properties and cut-off points in a Turkish adult population. Depress Anxiety 25(10):E104–E110. 10.1002/da.20371 17876817 10.1002/da.20371

[39] Tiftikçioğlu, B., Multiple Sclerosis Functional Composite (MSFC): scoring ınstructions. Noro Psikiyatr Ars, 2018. **55**(Suppl 1): p. S46-s48. 10.29399/npa.23330 10.29399/npa.23330 PMC6278631 30692855

[40] Sarihi S et al (2021) Toxic heavy metal concentrations in multiple sclerosis patients: a systematic review and meta-analysis. Excli j 20:1571–1584. 10.17179/excli2021-3484 34924905 10.17179/excli2021-3484 PMC8678057

[41] Bredholt M. and Frederiksen JL. 2016 Zinc in multiple sclerosis: a systematic review and meta-analysis. ASN Neuro, 2016. **8**(3). 10.1177/1759091416651511 10.1177/1759091416651511 PMC4904428 27282383

[42] Aliomrani M et al (2016) Blood concentrations of cadmium and lead in multiple sclerosis patients from Iran. Iran J Pharm Res 15(4):825–833 28243279 PMC5316261

[43] Janghorbani M., et al. Trace elements in serum samples of patients with multiple sclerosis. 2017. 10.30958/ajh.4-2-3

[44] Stojsavljević A et al (2024) Essential trace element levels in multiple sclerosis: bridging demographic and clinical gaps, assessing the need for supplementation. J Trace Elem Med Biol 83:127421. 10.1016/j.jtemb.2024.127421 38452433 10.1016/j.jtemb.2024.127421

[45] Forte G et al (2005) Quantification of chemical elements in blood of patients affected by multiple sclerosis. Ann Ist Super Sanita 41(2):213–216 16244395

[46] de Oliveira M et al (2020) A preliminary study of the concentration of metallic elements in the blood of patients with multiple sclerosis as measured by ICP-MS. Sci Rep 10(1):13112. 10.1038/s41598-020-69979-9 32753601 10.1038/s41598-020-69979-9 PMC7403292

[47] Almerud P et al (2021) Cadmium, total mercury, and lead in blood and associations with diet, sociodemographic factors, and smoking in Swedish adolescents. Environ Res 197:110991. 10.1016/j.envres.2021.110991 33705767 10.1016/j.envres.2021.110991

[48] Felicetti F et al (2025) Improvement of measured and perceived disability in overweight patients with multiple sclerosis trough different patterns of Mediterranean hypocaloric diet. Mult Scler Relat Disord 94:106271. 10.1016/j.msard.2025.106271 39823692 10.1016/j.msard.2025.106271

[49] Yigit P et al (2021) The relationship between cognition, depression, fatigue, and disability in patients with multiple sclerosis. Ir J Med Sci 190(3):1129–1136. 10.1007/s11845-020-02377-2 33006048 10.1007/s11845-020-02377-2

[50] Patti F et al (2009) Cognitive impairment and its relation with disease measures in mildly disabled patients with relapsing–remitting multiple sclerosis: baseline results from the Cognitive Impairment in Multiple Sclerosis (COGIMUS) study. Mult Scler J 15(7):779–788. 10.1177/1352458509105544 10.1177/1352458509105544 19542262

[51] Petracca M et al (2024) Telerehabilitation and onsite rehabilitation effectively improve quality of life, fatigue, balance, and cognition in people with multiple sclerosis: an interventional study. Front Neurol 15:1394867. 10.3389/fneur.2024.1394867 39175758 10.3389/fneur.2024.1394867 PMC11338795

[52] Boeschoten RE et al (2017) Prevalence of depression and anxiety in multiple sclerosis: a systematic review and meta-analysis. J Neurol Sci 372:331–341. 10.1016/j.jns.2016.11.067 28017241 10.1016/j.jns.2016.11.067

[53] Brenner P, Piehl F (2016) Fatigue and depression in multiple sclerosis: pharmacological and non-pharmacological interventions. Acta Neurol Scand 134(S200):47–54. 10.1111/ane.12648 27580906 10.1111/ane.12648

[54] Krikorian R et al (2010) Improved cognitive-cerebral function in older adults with chromium supplementation. Nutr Neurosci 13(3):116–122. 10.1179/147683010X12611460764084 20423560 10.1179/147683010X12611460764084

[55] Islam GMR et al (2022) Hair, serum and urine chromium levels in children with cognitive defects: a systematic review and meta-analysis of case control studies. Chemosphere 291:133017. 10.1016/j.chemosphere.2021.133017 34813844 10.1016/j.chemosphere.2021.133017 PMC8792285

[56] Yamamoto S et al (2014) Kinematic gait analysis and lactation performance in dairy cows fed a diet supplemented with zinc, Manganese, copper and cobalt. Anim Sci J 85(3):330–335. 10.1111/asj.12141 24206275 10.1111/asj.12141

